# Healthy Snack Project: Improving Healthy Choices through Multidisciplinary Food Education Actions

**DOI:** 10.3390/nu16020255

**Published:** 2024-01-14

**Authors:** Giuseppina Federici, Vincenzo Marcotrigiano, Erica Bino, Alberto Lovat, Angela Padoin, Gerardo Salerno, Pamela D’Incà, Christian Napoli, Sandro Cinquetti

**Affiliations:** 1 Prevention of Non-Communicable Diseases, Screening Programs and Health Promotion Service, Prevention Department, Local Health Authority “ULSS 1 Dolomiti”, 32100 Belluno, Italy; giuseppina.federici@aulss1.veneto.it (G.F.); alberto.lovat@aulss1.veneto.it (A.L.); angela.padoin@aulss1.veneto.it (A.P.); 2 Prevention Department, Local Health Authority “ULSS 1 Dolomiti”, 32100 Belluno, Italy; sandro.cinquetti@aulss1.veneto.it; 3 Epidemiology Service, Prevention Department, Local Health Authority “ULSS 1 Dolomiti”, 32100 Belluno, Italy; erica.bino@aulss1.veneto.it; 4 Department of Neurosciences, Mental Health and Sensory Organs “NESMOS”, Sapienza University of Rome, 00189 Rome, Italy; gerardo.salerno@uniroma1.it; 5 Communication Office, Local Health Authority “ULSS 1 Dolomiti”, 32100 Belluno, Italy; pamela.dinca@aulss1.veneto.it; 6 Department of Medical Surgical Sciences and Translational Medicine, Sapienza University of Rome, 00185 Rome, Italy; christian.napoli@uniroma1.it

**Keywords:** health promotion, school setting, break, mid-morning snack, healthcare workers, nutrition, public health

## Abstract

School is one of the main settings where it is useful to guarantee health promotion actions, as it is well known that diet and eating habits that are shaped in the early stages of life are maintained through adulthood. The objective of this study was to carry out the “Healthy Snack” project to promote nutritional education in primary schools in the Province of Belluno in the 2022–2023 school year, in which 925 students were enrolled, and to evaluate the intervention in terms of changes in eating habits during their school breaks. Following the workshops performed by the healthcare workers (HCWs), medals were awarded, taking into account the quality of the participating students’ mid-morning snacks, considering the food pyramid. The results collected in the annual survey period were related to the type and quantity of snacks consumed at school, and allowed students to gain a final score, comparing the period before and after the educational intervention to demonstrate the effectiveness of the actions promoted by HCWs and the increased nutritional quality of meals. In light of this evidence, public health strategies must continue to emphasize the importance of implementing health promotion interventions and actions aimed at children in order to prevent weight gain in this age group, and the potential development of cardiometabolic pathologies over their lifetime.

## 1. Introduction 

The relationship between health and diet is well known, and promoting correct eating habits in the preschool and school years is one of the strategic objectives of public health policies. A person’s diet is considered healthy when their usual eating patterns include an adequate intake of nutrients and sufficient, but not excessive, energy intake to satisfy their needs. Children around the world learn about and adopt eating habits from their parents and grandparents, teachers, siblings, and peers. Therefore, the home, community, and school environments provide important educational moments that help shape what, how much, when, and how children eat from birth through all stages of development. Globalization and urbanization have also contributed to changes in the domestic environment, which often influence the availability of food, as well as the commitment by parents and the way they eat, which have direct repercussions on their children [1]. 

Additionally, advances in food technology have led to increased availability of high-energy, low-nutrient foods (e.g., potato chips and sugary drinks) [2]. 

The consequences of these changes include overweight and obesity, which have become a real epidemic, not only in industrialized countries, but also in developing countries. Severe obesity continues to increase, representing significant costs in terms of indirect damage to organs and systems as well as the burden of disease [3,4,5,6]. 

Multiple strategies can be implemented at the personal, family, health system, community, and government level to help combat unhealthy eating habits, both individually and collectively [7]. Considering that most children spend a significant part of their day at school, many preventive interventions involve educational institutions in order to provide nutritional education and promote a healthy lifestyle, while encouraging greater physical activity [8].

Indeed, the school context is considered important for interventions on behaviors that can prevent obesity in children, since (i) primary education is compulsory for all children in most countries and reaches all students of different backgrounds; (ii) children spend a significant part of their daily lives at school, where they usually eat one or two meals a day; (iii) schools offer physical education lessons and provide opportunities for physical activity including during breaks; (iv) school provides a structured environment where interventions can be easily applied or adapted; (v) teachers can reach many children in a short time and with a small number of interventions; and (vi) teaching staff can facilitate and contribute significantly to the implementation of interventions, thus increasing their sustainability [9]. 

Recent studies show that an adequate number of daily meals, quantified as five meals per day, is associated with a lower risk of obesity in children; nevertheless, it has been shown that 10% to 30% of children and adolescents in 33 countries do not eat breakfast [10]. Snacks are generally eaten between main meals, often with the intention of reducing or preventing hunger until the next meal. The main problem is not only the frequency, but also the size of the portions and the types of foods consumed. Healthy food options, such as fruits and vegetables, should be promoted as snacks to avoid consumption of energy dense foods (chips, biscuits, sweets). Additionally, parents should be educated on healthy food choices and appropriate portion sizes and share this information with their children [11].

Scientific evidence shows that snacks represent approximately 27% of total daily calories, which is more than the calories consumed at breakfast (18%) and lunch (24%), but not dinner (31%). For children over the age of 4, more than 41% of daily snack calories come from foods such as chips, chocolate bars, soft drinks, fruit drinks, sugars, syrups, preserves, fats, and oils [12]. Mid-morning and mid-afternoon snacks are useful for controlling the appetite if they are of modest quantity (around 100 Kcal), thus avoiding the subsequent consumption of a meal that is too large. An adequate snack could consist of a piece of fruit, a cup of yogurt, or a spoonful of dried fruit, such as not to burden the day with food, but capable of controlling the appetite, thus avoiding a subsequent meal that is too large. In particular, in children, the choice of snack is not oriented towards the most appropriate healthy choices, which leads to consuming immediately available foods or the most palatable ones. This can lead to the choice of foods that are too high in energy intake and too rich in salt, sugar, or calories, with consequent poor appetite at the next meal and alteration of the hunger/satiety rhythm of the remaining meals, or with a general imbalance in the diet, which can easily lead to excess calories [13].

From a quantitative point of view, preventing excess weight is based on satisfying the child’s specific energy needs while ensuring compliance with the recommended amounts of macronutrients (12–15% protein, 30% lipids, 55–58% carbohydrates) and the total caloric distribution over five meals during the day (15% at breakfast, 5–10% at morning snack, 40% at lunch, 5–10% at afternoon snack, and 30–35% at dinner) [14].

To understand the dimensions of the phenomenon in children and the associated behaviors in our country, the Italian Ministry of Health has long promoted and financed the development of the Okkio alla Salute surveillance system, involving children attending the third class of primary school, meaning students from 8 to 9 years old. This system is coordinated by the National Center for Disease Prevention and Health Promotion of the Italian National Institute of Health, and is implemented in collaboration with the regions and the Italian Ministry of Education, University and Research. From the most recent survey results in 2019, it emerged that at a national level, 20.4% of children are overweight and 9.4% are obese, with severely obese children representing 2.4%. In particular, among those with incorrect eating habits, 55.3% have a plentiful mid-morning snack. 

Eating outside the three main meals—in other words, snacking—is part of the eating pattern of individuals at all life stages, but the positive or negative impact on health is mainly related to the nutritional content of the snack. Healthy fruit-based snacks and vegetables are commonly associated both with a better diet quality and a positive impact on the body weight; on the other hand, energy dense foods, such as cakes, buns, biscuits, and sugary drinks, contribute to increasing daily energy intake, which could lead to increased body weight and low nutrient quality [2]. The foundations of healthy eating habits, also depending on local traditions, have to satisfy the individual’s nutritional needs. Healthy nutrition from a modern perspective means protection from non-communicable diseases, promotion of health and longevity, and social and environmental sustainability. The main objective is the prevention of excess eating and obesity, which in Italy, especially in children, has worrying data. The Italian guidelines for correct nutrition are tailored on the basis of the Mediterranean dietary model, which has now acquired fame and honor throughout the world as the best model through which to combine health and well-being with sensorial satisfaction.

In the Veneto region, the data at the local level are better than at the national level: values are below the national average, with slightly lower values for overweight and obesity (19% overweight, 6.3% obesity). Furthermore, fewer than four out of ten children (38%) consume an adequate mid-morning snack; the majority of children (60%) have an inadequate snack; and 2% do not have one at all.

At the provincial level, 1% of children are severely obese, 2.6% are obese, and 22.4% are overweight; 37.2% have an inadequate snack, and 3.1% do not [15].

The Local Health Authority ULSS 1 Dolomiti includes the territory of the province of Belluno (3678 km²), being at the first place in terms of surface area among the provinces of the Veneto region. The Belluno area is made up of 61 municipalities for a total of 197,751 inhabitants, of which 96,528 (48.8%) are men and 101,223 are women (51.2%), with a constant decrease over the years. From 2017 to 2023 the number of people residing in the territory of ULSS 1 Dolomiti decreased by approximately 2%. The provincial population density is 264.6 inhabitants/km². The structure of the population is almost stable over the years, with 11% of people between 0 and 14 years, 61% between 15 and 64 years, and 28% over 65. In the province of Belluno there are 7269 children between 6 and 10 years old (the target age of our investigation), specifically 3721 males and 3548 females. Since 2018, the birth rate has been stable, at around 6‰, while the mortality rate has grown by two points, going from 12.3‰ to 14.2‰. The migratory balance is increasing from 3.3‰ in 2018 to 3.9‰ in 2022. Life expectancy at birth, which appears to be constant over the years, is 82.3 years, 79.9 for men and 84.9 for women. The population aging is one of the most important demographic aspects. Since 2018, the average age of the population has increased from 47.3 to 48.5 years and is approximately 2 years higher than the regional average, while the old age index (percentage ratio between the number of people over 65 and the number of young people up to 14 years old) has gone from 222.8 to 254. The trend towards population aging will also continue in the coming years, with a forecast for 2030 having an old age index of 303.8. Currently, in the territory of the ULSS 1 Dolomiti there are 22 comprehensive institutes and didactic circles related to primary schools, considered as school aggregation units, for a total of 86 complexes (buildings). There are 428 classes belonging to the buildings, for a total number of students equal to 6691.

The objective of the study was to describe the “Healthy Snack” project, a health promotion project focused on correct nutrition in childhood, and to evaluate the changes in the choice of mid-morning snacks made at school, following nutritional education interventions implemented in the 2022–2023 school year by the Prevention Department of ULSS 1 Dolomiti at primary schools in the Belluno area that joined the project.

## 2. Materials and Methods

“Healthy Snack” is a school project aimed at encouraging healthy eating and increasing basic physical activity among primary school pupils in the Province of Belluno. The food education intervention for children is aimed at encouraging and improving the creation of a positive relationship with healthy food, developing skills and knowledge in the area of nutrition, and adopting sustainable food practices. The project is included in the training offer proposed by ULSS 1 Dolomiti and intended for all primary schools in the local area.

### 2.1. Target Definition and Timing of Implementation

To define the target of the project, in-depth research was carried out in the fields of psychology and nutritional sciences, since, in the target age range, children are able to understand the complexity of the topics they are taught, but can still be influenced to dismantle unhealthy and unsustainable eating habits and establish new, healthy snack practices in everyday life. The project was promoted through a two-step training offer:

- In May 2022, the training offer of ULSS 1 Dolomiti aimed at schools was published and teachers were recruited.
- In September 2022, a training event was held with teachers to ensure class participation by October for the whole 2022–2023 school year, ending in June 2023. After approval by the class council, the teachers enrolled their classes in the project and the competition.

### 2.2. Project Path

The design path was divided into the following macro-actions:

1. Signing of the co-responsibility agreement.
2. Carrying out the recording of snacks.
3. Holding a workshop on the topic of nutrition, aimed at each registered class, by healthcare workers (HCWs) of the Health Promotion Service of the Prevention Department of ULSS 1 Dolomiti.

**Figure 1:**
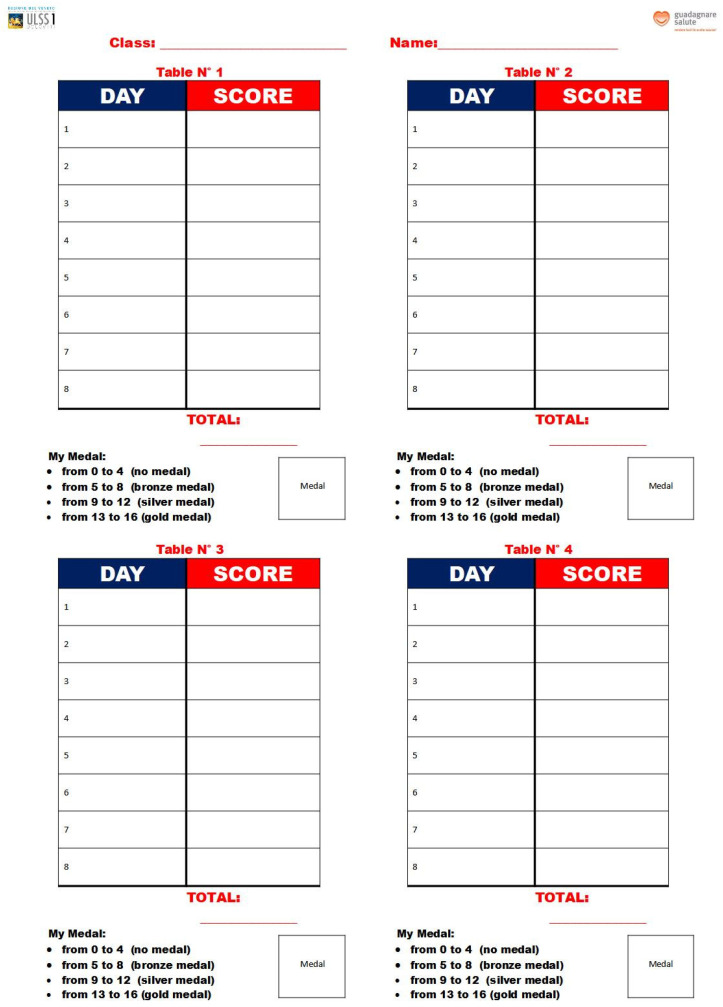
Table of scores for individual students.

**Figure 2:**
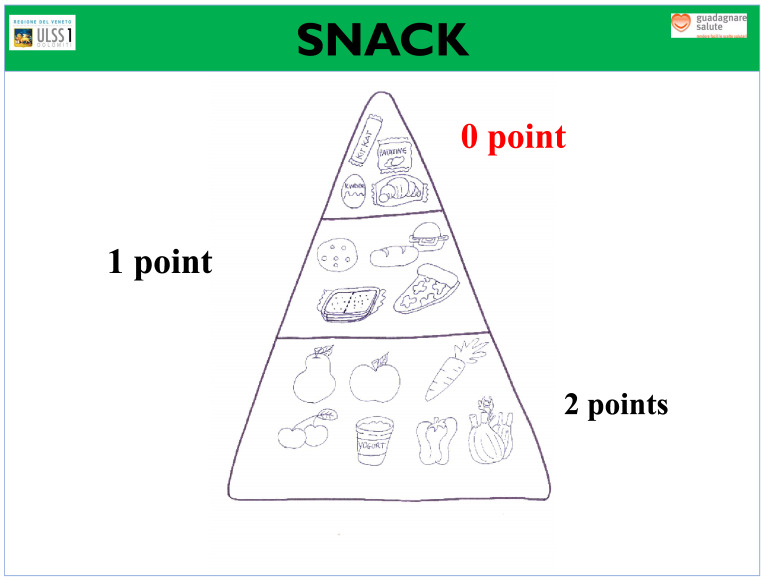
Scoring pyramid.

In detail, the process was divided into the following steps:

- Teachers, students, and parents signed the co-responsibility pact, valid for the entire school year. Teachers explained the benefits of a correct diet and constant, regular physical activity; promoted healthy eating habits at school and proposed that children also engage in games and physical activities at home; and supported students in recording their snacks. At the same time, students learned the information provided by teachers, brought a healthy snack to school, and committed to being more active every day (walking to school, taking the stairs, riding a bike, walking, helping their parents with housework) and spending as little time as possible with TV, video games, and computers. Students registered the snacks they consumed at school twice a week. In this context, parents can set a good example by providing their children with healthy snacks to take to school and offering fruits and vegetables at home (for breakfast, lunch, snack, and dinner), and organizing family exercise activities in their free time (walks, bike rides, etc.).
- Using a specific table that was provided for them, students recorded the snacks they consumed at school twice a week, for a total of 32 registrations for each participating student during the school year (Figure 1). On days randomly chosen by the teacher to carry out self-assessment, children filled out their own table by entering their score in relation to the snacks they consumed according to the food pyramid (Figure 2). The daily score was assigned as follows:
  ▪ No snack: 0 points.
  ▪ Inadequate snack (excessive portions of carbohydrates, sweet/savory snacks, packaged snacks): 0 points.
  ▪ Partially adequate snack (small sandwich, crackers, homemade biscuits): 1 point.
  ▪ Adequate snack (fruit, vegetables, or yogurt): 2 points.

**Figure 3:**
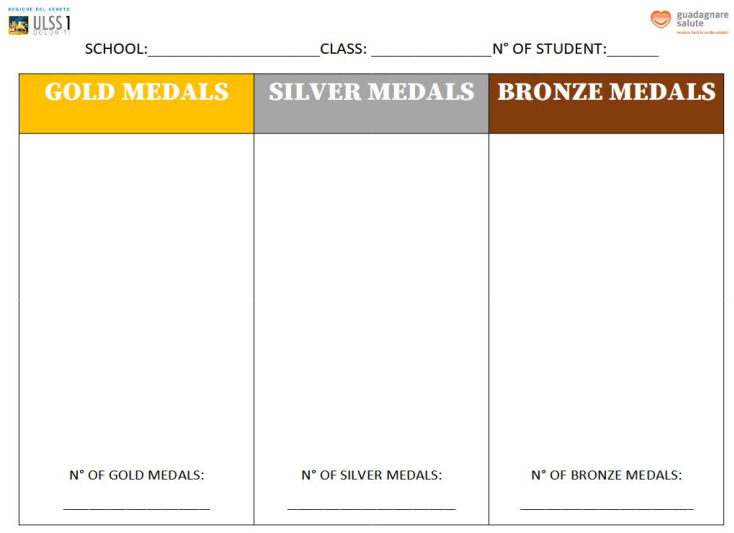
Class medal collection form.

Every 8 recordings, using the specially structured tables, students were asked to add up their score. Based on the total scores, an intermediate medal was given to each student: 0 to 4 points for inadequate snacks, no medal; 5 to 8 points for partially adequate snacks, bronze medal; 9 to 12 points for almost fully adequate snacks, silver medal; and 13 to 16 points for fully adequate snacks, gold medal. The gold medal score represented the summation of ideal snacks in terms of quantity, energy, and nutritional quality. The silver medal represented an intermediate score, as the snacks, although adequate in terms of energy, did not appear to be adequate in terms of nutritional quality. The bronze medal was awarded for a lower score, as the snacks were found to be less than adequate. At the end of the 32 surveys carried out during the entire project (corresponding to approximately 6 months), each student may have collected up to 4 medals, which could be entered on a class medal table (Figure 3). 

**Figure 4:**
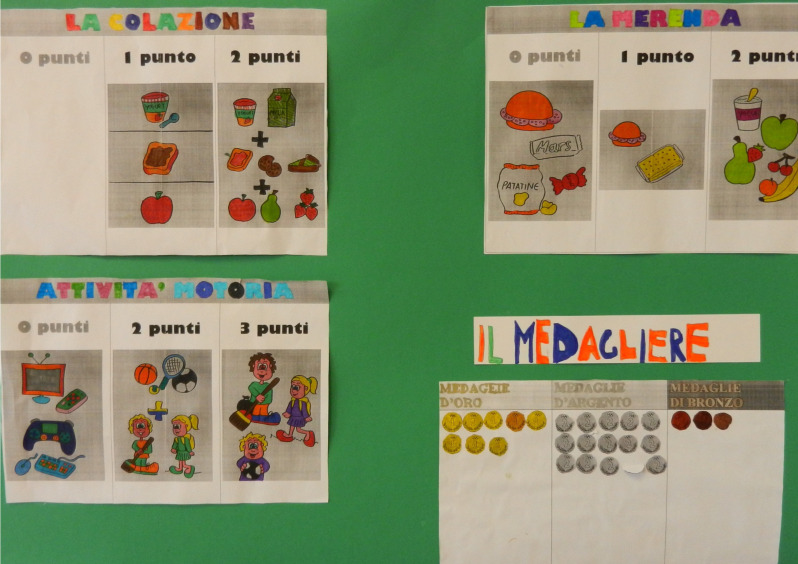
Class medal table at the end of the survey. In Italian language, *colazione* means breakfast, *merenda* means snack, *attività motoria* means physical activity, and *medagliere* means medal collection.

**Figure 5:**
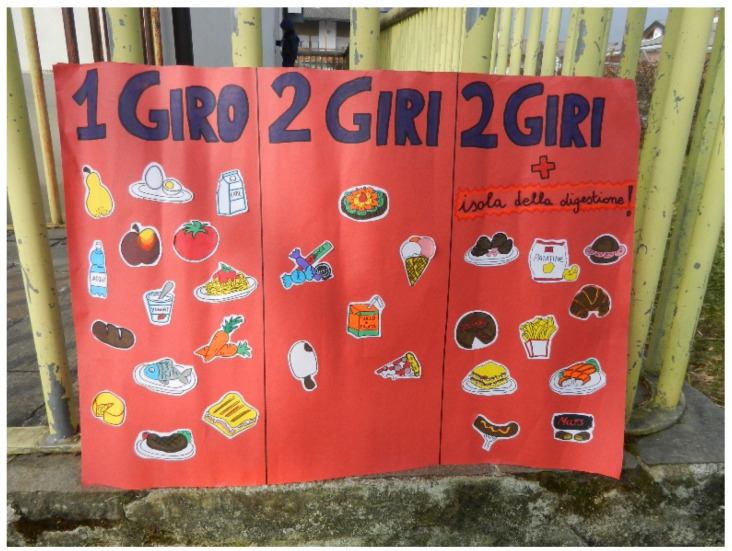
Poster representing the complexity of the digestive process. In Italian language, *giro* means round and *isola della digestione* means digestion island.

At the end of the individual surveys, all medals obtained by the students in each class were added up. The classes were rewarded in proportion to the number of pupils who collected the greatest number of medals (Figure 4).

c. A nutrition laboratory was held for each enrolled class during the school year, particularly in the months when the students joined the project. The workshops promoted correct nutrition and encouraged children to choose what and how to eat. The information was provided for children in a playful way, paying particular attention to the daily consumption of fruits and vegetables. The laboratories supported and integrated the activities carried out in class by the teachers.
  The laboratory activities were carried out by a pair of HCWs with different profiles: dietician, health visitor, social health educator, and environmental health officer. The five workshops, each lasting 2 h, were differentiated based on the ages of students and were structured as follows:
  ▪ Playground games workshop: intended for pupils attending the first classes of primary school. The HCWs introduced the children to the pleasure of experimenting with new group games and moving, preferably in the open air, through education using music, rhythm, and motor coordination.
  ▪ Creative–sensory laboratory: intended for pupils in the second year of primary school. Children had the opportunity to learn about fruits and vegetables through stories and sensory journeys.
  ▪ Laboratory on oral hygiene: intended for pupils in the third year of primary school. This laboratory was aimed at helping children understand the importance of oral hygiene, and described friendly, hostile, and junk foods.
  ▪ Scientific laboratory: intended for pupils in the fourth grade of primary school. This laboratory was aimed at transmitting basic information to children about macronutrients, their function within the body, and the division and composition of meals during the day.
  ▪ Motor skills workshop: intended for pupils in the fifth grade of primary school. This workshop was aimed at helping children understand the path of food within the body through motor activities (Figure 5). The main objectives were achieved by observing and reflecting on the methods of digestion of foods.

During the 2022–2023 school year, 59 classes with a total of 925 students joined the “Healthy Snack” project. The sample of 925 students is representative of the 6691 eligible children in the Belluno province, with a confidence level of 95%. The average age of the children participating in the project is 8.04 years. The total and enrollment number of classes and students are detailed in Table 1.

Following the approximately two-hour intervention directly performed by the healthcare workers, each class took about an hour to fill the tables and assign the medals, twice a week. In addition to this, each class was free to delve deeper into the concept of healthy eating and lifestyle (Mediterranean lifestyle, consumption of fruit and vegetables, physical activity, and sleeping quality). No teacher or student abandoned the project, once started. 

The investigation was performed in accordance with the World Medical Association Declaration of Helsinki and did not include any experiments involving human or biological human samples, nor research on identifiable human data. Regardless, the study protocol was approved, also with regard to ethical issues, by the Local Health Authority ULSS 1 Dolomiti (approval no. DP0109_2022).

## 3. Results

At the initial time T0, the percentage of children who had an adequate snack in ULSS 1 Dolomiti was 59.7%. According to the criteria defined in our study, this was calculated by considering the sum of gold and silver medals assigned to each student as an inclusion criterion determining a “healthy” snack. At time T0, 2209 gold and silver medals were awarded. The percentage of children who had a healthy snack at time T1, intended as the endpoint of the project, was calculated by taking into account the 3172 total medals awarded and applying the following formula:

(Gold medals + Silver medals)/(Number of medals assignable) × 100 = (3172/3700) × 100 = 85.7%

Comparing the two percentages, we find a significant increase in children having healthy snacks. By applying the chi-square test, a *p*-value equal to 0.0001 is obtained, confirming the statistical significance of the increase (Table 2).

## 4. Discussion

Unhealthy eating habits and an unhealthy lifestyle are considered to be the main causes of obesity, especially in childhood [16,17]. In light of this, current public health strategies emphasize the importance of continuing to implement health promotion interventions aimed at children in primary schools. 

In particular, in ULSS 1 Dolomiti, the latest results of Okkio alla Salute surveillance, carried out in 2019, confirm a widespread pattern of incorrect eating habits among school-age children, with 40% of children having inappropriate mid-morning snacks (i.e., incorrect or none at all).

Our study analyzed the quality of snacks at school directly brought from home before and after a specifically structured educational intervention among 925 children participating in the project’s dedicated and inclusive training offered in schools by HCWs. Medals were assigned to children for the scores they obtained based on the types of snacks they chose, evaluated in two weekly surveys conducted over six months. The scores for gold and silver medals were considered adequate, as they respected the characteristics of the snack (in terms of energy and nutritional quality). During the 2022–2023 school year, compared to the initial survey, it is found that 59.7% of children had adequate snacks; these data are in line with the latest results of the Okkio alla Salute survey, referring to the entire ULSS 1 Dolomiti. The percentage of snacks considered adequate after the educational intervention was 85.7%, and this difference was statistically significant. 

In our country, other studies have been performed demonstrating the importance of planning integrated and multisectoral actions in school programs to promote correct eating habits, suggesting that educational interventions in the school setting could be a key strategy in the prevention of obesity, helping to achieve the following objectives: (i) reduction in food consumption or energy intake; (ii) increased consumption or preference for fruits and vegetables; (iii) reduction in sugar consumption; and (iv) increase in nutritional knowledge. The results that emerged in the pilot phase of our study reinforce the evidence described in the sector studies [18,19,20]. 

The attention paid to this age group by the Health Promotion Service of the Prevention Department has therefore demonstrated how the intervention carried out by HCWs can have a significant impact on the choices made by children, with the hope that eating habits can be maintained over time and demonstrated through further studies.

School is primarily an educational setting, but it is also a place for nutrition, and interventions conducted by a multi-professional team represent a valid strategy [21].

In addition to the family home, school is a place where children can learn about healthy lifestyles, including healthy foods available in the school canteen and physical activity. Children spend a significant portion of their daily time in school and are influenced by their physical and social environment, such as school health policies, educational and nutritional support, and physical education. In this regard, school is an ideal environment to reach as many children as possible, regardless of gender, socio-economic status, or social background, and is also a favorable environment for reducing health inequalities [22]. Along with lunch in the school canteen, the mid-morning snack is one of the times when eating takes place within the school [23]. Unlike lunch and the mid-afternoon snack, the morning snack is a common experience for children and is a “bridge” food moment between home and school; as such, healthy behaviors can be promoted by also involving families. Our project acted precisely on these aspects, showing a correct action strategy. Moreover, since the project did not provide direct access to healthy snacks, family economic conditions may have influenced, both positively and negatively, some choices attributable to the subsequent awarding of medals. 

Furthermore, it is widely demonstrated that the media, particularly commercials, shape knowledge, attitudes, and preferences, and influence practices related to food; in particular, this manifests as increased intake of snacks and calories, and decreased consumption of fruits and vegetables. Currently, marketing for unhealthy foods is ubiquitous. Foods rich in fat, salt, and sugar are heavily advertised on various media platforms, including digital platforms; these ads are often aimed at children, such as on social media, and these foods can be bought almost everywhere. This may contribute to the obesity epidemic we are experiencing. Reducing the marketing of energy dense snacks to children and increasing the promotion of healthier foods, such as fruits and vegetables, can be an effective approach and may be necessary to improve children’s nutritional intake and reduce the risk that they will develop chronic diseases later in life [24]. In particular, European and American children are heavily exposed to mass media; depending on their age and taking into account the multiple uses of electronic media, recent reports show that children and young people aged 8 to 18 are exposed to more than 7 h of daily use. Among other things, foods eaten in front of the television represent approximately 20–25% of children’s daily energy intake [25].

A child’s growth environment has an important influence on the choice of a healthy lifestyle: the two major sources of influence that make up this setting include family and school. Families play a priority role encouraging attitudes that support the positive vision of healthy eating and dietary practices, as well as transmit important cultural and emotional connections with food. Constantly reinforcing healthy living, it can have a huge influence on a child’s lifestyle activities, including selecting foods to eat, eating patterns, and physical activity. When parents are active as part of their lifestyle and routinely eat healthy foods, they serve as role models for their children, who engage in similar healthy behaviors. Children can learn indirectly from their parents by watching them while food shopping and by observing food cooking at home. Children spend a significant portion of their daily time at school and are influenced by their physical and social environment, such as school health policies, education, nutritional support, and physical education. In this regard, schools are an ideal environment to reach as many children as possible, regardless of gender, socio-economic status, or social background, and represent a favorable environment for reducing health inequalities [22]. This turns out to be one the main strengths of our investigation.

Our study, although limited to the territory of a single local health authority, proved to be effective at aiming at the right target with the right modality at the local level, allowing us to go beyond monitoring national health. In fact, Okkio alla Salute is a population survey based on epidemiological investigations of representative samples of the population repeated on a regular basis. It is therefore a collection oriented toward more standardized information, and mainly investigates modifiable risk factors through the use of simple tools and procedures, acceptable by operators and citizens and sustainable by health systems. Okkio alla Salute selects children from the third class of primary school because growth at the age of 8 is still minimally influenced by puberty, and children are able to answer some simple questions reliably. 

However, the subscription of the classes that participated in the “Healthy Snack” project could be attributable to the voluntary participation in the training offer; therefore, the particular sensitivity of the teachers on the topic of food education, where it is assumed that the children already carry out ongoing activities in collaboration with teachers and external bodies, could have affected the outcomes. Moreover, our study did not involve the presence of a control group, and the improvement observed in the comparison between T0 and T1 is indirectly attributable to the increase in the number of medals obtained. Furthermore, Okkio alla Salute exclusively targets children in the third class of primary school, while our study examined all children participating in the “Healthy Snack” project from the first to the fifth classes of primary school, with a larger degree of heterogeneity. Finally, the results derived from the Okkio alla Salute survey in 2022, following the pandemic period, will be indispensable for evaluating any expected deviations in terms of eating habits and weight gain.

## 5. Conclusions

The effectiveness of an encouraging nutritional intervention program as a strategy to guide the purchase and consumption of healthier foods is determined by numerous variables, including the specific setting, the target population, and the multiple professionals involved. Intervening in the school context, where most children spend a significant part of the day, is advantageous and can have a significant impact on eating behaviors that can be learned and maintained over time, contributing to a reduced prevalence of overweight and obesity, and consequently preventing cardiometabolic pathologies throughout life.

## Figures and Tables

**Table 1:** Number of classes and students in total and enrolled.

Primary School Classes	Number of Classes Enrolled	Number of Students Enrolled	Number of Total Classes	Number of Total Students
First class	11	160	81	1250
Second class	13	195	80	1283
Third class	12	187	91	1333
Fourth class	12	213	85	1368
Fifth class	11	170	91	1457
Total	59	925	428	6691

**Table 2:** Percentage of medals awarded at times T0 and T1.

	Medals at T0 (%)	Medals at T1 (%)	*p*-Value
Assigned Medals	2209 (59.7)	3172 (85.7)	0.0001

## Data Availability

Data are contained within the article.
